# Items

**Published:** 1910-04

**Authors:** 


### ITEMS.

---

THE Denver Chemical Manufacturing Company, sometime ago brought suit against the Colorado Chemical Company for marketing an imitation of antiphlogistine which is manufactured and distributed by the former company. In the United States Circuit Court, District of Kansas, a decision was rendered recently, granting to the complainant all that was claimed in the bill. This will serve as another warning to imitators that the law relating to such matters cannot be violated with impunity.

---

BATTLE & CO., of St. Louis, have just issued No. 12 of their series of charts on dislocations. This series forms a valuable and interesting addition to any physician's library. They will be sent free of charge on application, and back numbers will also be supplied. If anyone has missed any of the numbers, write Battle & Co. for them before the supply is exhausted.

